# Myeloablative conditioning for allo-HSCT in pediatric ALL: FTBI or chemotherapy?—A multicenter EBMT-PDWP study

**DOI:** 10.1038/s41409-020-0854-0

**Published:** 2020-03-17

**Authors:** Andre Manfred Willasch, Christina Peters, Petr Sedláček, Jean-Hugues Dalle, Vassiliki Kitra-Roussou, Akif Yesilipek, Jacek Wachowiak, Arjan Lankester, Arcangelo Prete, Amir Ali Hamidieh, Marianne Ifversen, Jochen Buechner, Gergely Kriván, Rose-Marie Hamladji, Cristina Diaz-de-Heredia, Elena Skorobogatova, Gérard Michel, Franco Locatelli, Alice Bertaina, Paul Veys, Sophie Dupont, Reuven Or, Tayfun Güngör, Olga Aleinikova, Sabina Sufliarska, Mikael Sundin, Jelena Rascon, Ain Kaare, Damir Nemet, Franca Fagioli, Thomas Erich Klingebiel, Jan Styczynski, Marc Bierings, Kálmán Nagy, Manuel Abecasis, Boris Afanasyev, Marc Ansari, Kim Vettenranta, Amal Alseraihy, Alicja Chybicka, Stephen Robinson, Yves Bertrand, Alphan Kupesiz, Ardeshir Ghavamzadeh, Antonio Campos, Herbert Pichler, Arnaud Dalissier, Myriam Labopin, Selim Corbacioglu, Adriana Balduzzi, Jacques-Emmanuel Galimard, Peter Bader, on behalf of the EBMT Paediatric Diseases Working Party

**Affiliations:** 1 Division for Stem Cell Transplantation and Immunology, Department for Children and Adolescents, University Hospital, Goethe University Frankfurt, Frankfurt, Germany; 2 St. Anna Children’s Hospital, Department of Pediatrics, Medical University of Vienna, Vienna, Austria; 3 Department of Pediatric Hematology and Oncology, University Hospital Motol, Prague, Czech Republic; 4 Department of Pediatric Hemato–Immunology, Hôpital Robert Debré and Université de Paris, Paris, France; 5 Stem Cell Transplant Unit, Aghia Sophia Children’s Hospital, Thivon and Papadiamantopoulou, Athens, Greece; 6 Department of Pediatric Hematology and Stem Cell Transplantation Unit, Medicalpark Antalya Hastanesi, Antalya, Turkey; 7 Department of Pediatric Oncology, Hematology and Transplantology, University of Medical Sciences, Poznan, Poland; 8 Department of Pediatrics, Leiden University Medical Center, Leiden, The Netherlands; 9 Hematology–Oncology Unit “Lalla Seràgnoli”, Department of Pediatrics, University of Bologna, Bologna, Italy; 10 Pediatric Cell Therapy Research Center, Tehran University of Medical Sciences, Tehran, Iran; 11 Department of Pediatrics, Copenhagen University Hospital, Rigshospitalet, Copenhagen, Denmark; 12 Department of Pediatric Hematology and Oncology, Oslo University Hospital, Oslo, Norway; 13 Department for Pediatric Hematology and Hemopoietic Stem Cell Transplantation, Central Hospital of Southern Pest, National Institute of Hematology and Infectious Diseases, United St. Istvan and St. László Hospital, Budapest, Hungary; 14 Centre Pierre et Marie Curie, Service Hématologie Greffe de Moelle, Alger, Algeria; 15 Servicio de Hematologia y Oncologia Pediátricas, Hospital Universitario Vall d’Hebron, Barcelona, Spain; 16 BMT Department, Russian Children’s Hospital, Moscow, Russia; 17 Department of Pediatric Hematology and Oncology and Research Unit EA 3279, Timone Enfants Hospital, AP-HM and Aix-Marseille University, Marseille, France; 18 Dipartimento di Onco-Ematologia e Terapia Cellulare e Genica, IRCCS Ospedale Pediatrico Bambino Gesù, Rome, Italy; 19 Dipartimento Materno-Infantile e Scienze Urologiche, Sapienza University of Rome, Rome, Italy; 20 Division of Stem Cell Transplantation and Regenerative Medicine, Department of Pediatrics, School of Medicine, Stanford University, Stanford, CA USA; 21 Great Ormond Street Hospital for Children NHS Foundation Trust, London, UK; 22 Department of Pediatric Hematology and Oncology, Cliniques Universitaires Saint-Luc, Brussels, Belgium; 23 Department of Bone Marrow Transplantation, Hadassah-Hebrew University Medical Center, Jerusalem, Israel; 24 Division of Stem Cell Transplantation, Children’s Research Center (CRC), University Children’s Hospital, Zurich, Switzerland; 25 Republic Clinical Research Centre for Pediatric Oncology and Hematology, Minsk, Belarus; 26 BMT Unit, Department of Pediatric Hematology and Oncology, Comenius University Medical School, Limbová, Bratislava, Slovak Republic; 27 Division of Pediatrics, CLINTEC, Karolinska Institutet and Pediatric Hematology, Immunology and HCT, Astrid Lindgren Children’s Hospital, Karolinska University Hospital, Stockholm, Sweden; 28 Center of Pediatric Oncology and Hematology, Vilnius University, Vilnius, Lithuania; 29 Tartu University Hospital, Tartu, Estonia; 30 Department of Haematology, Internal Clinic, University Hospital Centre, Zagreb, Croatia; 31 Paediatric Onco-Haematology, City of Science and Health of Turino, Regina Margherita Children’s Hospital, Torino, Italy; 32 Department of Pediatric Hematology and Oncology, Collegium Medicum, Nicolaus Copernicus University Torun, Bydgoszcz, Poland; 33 BMT-Unit, University Medical Centre Utrecht Pediatrics, Utrecht, The Netherlands; 34 Department of Hematology, Child Welfare Center, Borsod County Teaching Hospital, Miskolc, Hungary; 35 Bone Marrow Transplant Program, Instituto Portugues Oncologia, Lisbon, Portugal; 36 Raisa Gorbacheva Memorial Research Institute for Pediatric Oncology, Haematology and Transplantation, Saint Petersburg State Medical I.P. Pavlov University, Saint Petersburg, Russia; 37 Department of Pediatrics, Onco-Hematology Unit, Geneva University Hospital, Geneva University, Geneva, Switzerland; 38 Hospital for Children and Adolescents, University of Helsinki, Helsinki, Finland; 39 Department of Pediatric Hematology Oncology, King Faisal Specialist Hospital & Research Center, Riyadh, Saudi Arabia; 40 Department of Bone Marrow Transplantation, Oncology and Hematology, Cape of Hope Medical Center, Wroclaw Medical University, Wroclaw, Poland; 41 University Hospitals Bristol NHS Foundation Trust, Bristol, UK; 42 Department of Pediatric Hematology and BMT, IHOP and Claude Bernard University, Lyon, France; 43 Department of Pediatric Hematology–Oncology, School of Medicine, Akdeniz University, Antalya, Turkey; 44 Hematology-Oncology and BMT Research, Shariati Hospital, Tehran, Iran; 45 Bone Marrow Transplantation Service, Portuguese Institute of Oncology, Porto, Portugal; 46 EBMT Paediatric Diseases Working Party, Paris, France; 47 EBMT Paris Study Office, Paris, France; 48 Department of Pediatric Hematology, Oncology and Stem Cell Transplantation, University of Regensburg, Regensburg, Germany; 49 Bone Marrow Transplantation Unit, Pediatric Department of Milano-Bicocca University, Fondazione Monza e Brianza per il Bambino e la sua Mamma Foundation, Monza, Italy

**Keywords:** Stem-cell research, Acute lymphocytic leukaemia

## Abstract

Although most children with acute lymphoblastic leukemia (ALL) receive fractionated total body irradiation (FTBI) as myeloablative conditioning (MAC) for allogeneic hematopoietic stem cell transplantation (allo-HSCT), it is an important matter of debate if chemotherapy can effectively replace FTBI. To compare outcomes after FTBI versus chemotherapy-based conditioning (CC), we performed a retrospective EBMT registry study. Children aged 2–18 years after MAC for first allo-HSCT of bone marrow (BM) or peripheral blood stem cells (PBSC) from matched-related (MRD) or unrelated donors (UD) in first (CR1) or second remission (CR2) between 2000 and 2012 were included. Propensity score weighting was used to control pretreatment imbalances of the observed variables. 3.054 patients were analyzed. CR1 (1.498): median follow-up (FU) after FTBI (1.285) and CC (213) was 6.8 and 6.1 years. Survivals were not significantly different. CR2 (1.556): median FU after FTBI (1.345) and CC (211) was 6.2 years. Outcomes after FTBI were superior as compared with CC with regard to overall survival (OS), leukemia-free survival (LFS), relapse incidence (RI), and nonrelapse mortality (NRM). However, we must emphasize the preliminary character of the results of this retrospective “real-world-practice” study. These findings will be prospectively assessed in the ALL SCTped 2012 FORUM trial.

## Introduction

Most children with acute lymphoblastic leukemia (ALL) above 2 years of age being candidate to be treated with allogeneic hematopoietic stem cell transplantation (allo-HSCT) receive myeloablative conditioning (MAC) with a fractionated total body irradiation (FTBI)-containing regimen [1–17]. It is an important matter of debate if chemotherapy can effectively replace FTBI. Due to the known late effects associated with the use of FTBI, which include endocrine complications (growth impairment, hypothyroidism, and delayed onset of puberty), infertility, cognitive impairment, cataracts, and an increased risk for secondary malignancies, avoidance of FTBI in the preparation of allo-HSCT is desirable [18–23]. To date, it has not been shown that FTBI can be successfully replaced by chemotherapy during conditioning for pediatric ALL [2, 3, 5, 24–26].

To compare outcomes of FTBI versus chemotherapy-based conditioning (CC) in childhood ALL, we performed this international retrospective registry-based study. The study was initiated and conducted on behalf of the Paediatric Diseases Working Party of the European Society for Blood and Marrow Transplantation (EBMT). The primary endpoint was leukemia-free survival (LFS). Overall survival (OS), relapse incidence (RI), nonrelapse mortality (NRM), and incidence of acute graft versus host disease (aGvHD) and chronic GvHD (cGvHD) were the secondary endpoints.

## Patients and methods

Children and adolescents aged between 2 and 18 years undergoing a first allo-HSCT for ALL in first (CR1) or second complete remission (CR2) after MAC with either bone marrow (BM) or peripheral blood stem cells (PBSC) from either a matched-related (MRD) or unrelated donor (UD) between 2000 and 2012 were included in the study. This observation period was chosen in order to obtain a reasonable time of follow-up (FU). Moreover, the prospective international randomized ALL SCTped 2012 FORUM trial was started in 2013 and is still recruiting patients. Data were obtained from the EBMT database ProMISe (Project Manager Internet Server) and analyzed in the EBMT study office in Paris, France. The study was performed in accordance with the Declaration of Helsinki. The local institutional review board at each participating site approved the allo-HSCT procedures. Patients and/or their legal guardians gave written informed consent to use clinical data and research participation. All authors had access to the primary clinical data.

### Statistical analysis

The study population was divided into two groups (patients in CR1 and CR2). Patients’ demographic and clinical characteristics were summarized using the median and interquartile range for continuous variables and counts and percentages for categorical variables. Preparative regimens were FTBI versus CC. For both remission groups the two conditioning regimens were compared using Fisher’s exact test or *χ*² test for categorical variables and Wilcoxon rank sum test for continuous variables [27].

Median FU was calculated using the reverse Kaplan–Meier method. The primary endpoint was LFS defined as the probability of being alive and free of disease at any point in time. Thus, death or disease relapse was treated as events. Patients alive and free of disease at their last FU were censored [28, 29]. OS was defined as the probability of survival irrespective of the disease state at any point in time. Patients alive at their last FU were censored. RI was defined as the probability of having experienced a relapse. Death without experiencing a relapse was the competing event. NRM was defined as the probability of dying without previous occurrence of a relapse, which was considered as competing event. Incidences of aGvHD (grade III–IV), cGvHD, and extensive cGvHD were defined as first event of aGvHD (grade III–IV), cGvHD, and extensive cGvHD, respectively. Death and relapse were considered as competing events. OS, RI, NRM, and incidence of acute and cGvHD were secondary endpoints [30].

The inverse probability weighting (IPW) method using the propensity score was used to calculate weights and adjust for confounding factors between the treatment groups [31]. Confounding factors considered age at allo-HSCT, year of allo-HSCT, time from diagnosis to allo-HSCT, cytomegalovirus (CMV) serology, stem cell source, and sex mismatch (female to male versus other).

The weighted Kaplan–Meier method was used to estimate the standardized probability of survival for LFS and OS, and the weighted cumulative incidence function was used to calculate cumulative incidence of relapse (RI), NRM, acute, and cGvHD [27–30]. *P* values to evaluate survival differences between the two conditioning regimens were calculated using a weighted proportional hazards Cox model including center as a random effect [32]. Results were expressed as weighted probabilities, weighted cumulative incidences, and hazard ratio with their 95% confidence intervals (95% CI). All tests were two-sided. The type 1 error rate was fixed at 0.05 for determination of factors associated with time to event. Analyses were performed using the R statistical software, Version 3.4.3 (R Development Core Team, Vienna, Austria). Weights were calculated using the twang R package [33]. The date of analysis was October 1, 2018.

## Results

### Characteristics of study patients

3.054 pediatric patients from European and non-European EBMT centers in 45 countries were included. Between 2000 and 2012, 2.630 patients received a FTBI-based and 424 patients received a chemotherapy-based MAC before allo-HSCT. 1.498 patients (49%) were transplanted in CR1 and 1.556 (51%) in CR2. In the CR1 cohort, median FU was 6.8 years (FTBI group) and 6.1 years (CC group), while in the CR2 cohort, median FU was 6.2 years in the FTBI and in the CC group. In both remission groups, the two conditioning groups differed significantly with regard to age at allo-HSCT, year of allo-HSCT, time from diagnosis to allo-HSCT, stem cell source, and CMV serology (donor/patient, Table 1). These confounding factors and the different sizes of the two conditioning groups requested adjustment by the inverse IPW method (propensity score, see “Statistical analysis”).

**Table 1:** Patient characteristics.

Patient characteristics		CR1				CR2			
		Total	CC^a^	FTBI^b^		Total	CC^c^	FTBI^d^	
Variable	Statistic/level	*n* = 1498	*n* = 213	*n* = 1285	*P* value	*n* = 1556	*n* = 211	*n* = 1345	*P* value
Follow-up (years)	Median [95% CI]	6.3 [6.2–6.6]	6.1 [6.0–6.3]	6.8 [6.4–6.7.0]	<0.0001	6.2 [6.0–6.5]	6.2 [6.2–6.5]	6.2 [6.0–6.8]	0.36
Age (years)	Median (range) [IQR]	11.2 (2.0–18.0) [7.1–15.4]	8.8 (2.0–18.0) [4.3–14.6]	11.5 (2.0–18.0) [7.7–15.5]	<0.0001	9.6 (2.0–18.0) [6.8–13.6]	8.7 (2.0–17.9) [4.7–12.9]	9.7 (2.1–18.0) [6.9–13.7]	0.0003
Patient sex	Male	988 (66.1%)	134 (63.8%)	854 (66.5%)	0.44	1034 (66.6%)	146 (69.9%)	888 (66.1%)	0.28
	Female	506 (33.9%)	76 (36.2%)	430 (33.5%)		519 (33.4%)	63 (30.1%)	456 (33.9%)	
	Missing	4	3	1	–	3	2	1	–
Immunophenotype	B	908 (64.2%)	146 (70.2%)	762 (63.1%)	0.13	1157 (80.6%)	135 (70.0%)	1022 (82.3%)	<0.0001
	T	448 (31.6%)	56 (26.9%)	392 (32.5%)		204 (14.2%)	32 (16.6%)	172 (13.8%)	
	Other	59 (4.2%)	6 (2.9%)	53 (4.4%)		74 (5.2%)	26 (13.4%)	48 (3.9%)	
	Missing	83	5	78	–	121	18	103	–
Year of HSCT	Median (range) [IQR]	2007 (2000–2012) [2004–2010]	2010 (2000–2012) [2007–2011]	2007 (2000–2012) [2004–2010]	<0.0001	2007 (2000–2012) [2004–2010]	2009 (2000–2012) [2005–2011]	2007 (2000–2012) [2004–2010]	<0.0001
Diagnosis to HSCT (months)	Median (range) [IQR]	6.7 (1.3–84.4) [5.5–8.3]	7.5 (2.0–78.8) [6.2–9.5]	6.6 (1.2–84.4) [5.5–8.2]	<0.0001	34.6 (1.3–159.7) [22.4–48.7]	30.6 (5.1–145.4) [20.1–47.1]	35.5 (1.3–159.7) [23.2–49.1]	0.02
Donor type	MRD	756 (50.5%)	109 (51.2%)	647 (50.3%)	0.82	672 (43.2%)	102 (48.3%)	570 (42.4%)	0.1
	UD	742 (49.5%)	104 (48.8%)	638 (49.7%)		884 (56.8%)	109 (51.7%)	775 (57.6%)	
Stem cell sources	BM	1041 (69.5%)	121 (56.8%)	920 (71.6%)	<0.0001	1064 (68.4%)	102 (48.3%)	962 (71.5%)	<0.0001
	PBSC	457 (30.5%)	92 (43.2%)	365 (28.4%)		492 (31.6%)	109 (51.7%)	383 (28.5%)	
Donor to patient sex mismatch	Female to male	374 (25.2%)	58 (27.4%)	316 (24.9%)	0.44	400 (25.8%)	63 (30.0%)	337 (25.2%)	0.14
	Other	1109 (74.8%)	154 (72.6%)	955 (75.1%)		1149 (74.2%)	147 (70.0%)	1002 (74.8%)	
	Missing	15	1	14	–	7	1	6	–
TBI dose (Gy)	Median (range) [IQR]	–	–	12.0 (7.5–16.0) [12.0–12.0]	–	–	–	12.0 (7.5–16.0) [12.0–12.0]	–
	Missing	–	–	263	–	–	–	294	–
Busulfan dose (mg/kgBW)	Median (range) [IQR]	–	16.0 (9.6–20.0) [12.8–17.6]	–	–	–	16.0 (9.6–20.0) [12.8–16.0]	–	–
	Missing	–	30	–	–	–	30	–	–
White blood count (cells/nl) at diagnosis	Median (range) [IQR]	45.0 (0.2–900.0) [8.0–157.7]	36.6 (0.3–862.0) [12.3–142.0]	46.0 (0.2–900.0) [8.0–159.1]	0.798	19.1 (0.1–672.0) [6.0–70.6]	26.0 (0.6–600.0) [6.9–96.8]	18.7 (0.1–672.0) [5.8–64.7]	0.22
	Missing	949	160	789	–	1100	160	940	–
CMV, donor/patient	−/−	419 (34.8%)	41 (22.5%)	378 (36.9%)	0.0003	426 (34.4%)	48 (25.2%)	378 (36.0%)	0.006
	−/+	228 (18.9%)	48 (26.4%)	180 (17.6%)		229 (18.5%)	37 (19.5%)	192 (18.3%)	
	+/−	122 (10.1%)	15 (8.2%)	107 (10.5%)		141 (11.4%)	18 (9.5%)	123 (11.7%)	
	+/+	436 (36.2%)	78 (42.9%)	358 (35.0%)		443 (35.7%)	87 (45.8%)	356 (34.0%)	
	Missing	293	31	262	–	317	21	296	–
Acute GvHD	No aGvHD	587 (40.6%)	112 (53.8%)	475 (38.3%)	–	550 (37.0%)	99 (48.3%)	451 (35.2%)	–
	Grade I	318 (22.0%)	27 (13.0%)	291 (23.5%)		321 (21.6%)	28 (13.7%)	293 (22.9%)	
	Grade II	366 (25.3%)	39 (18.8%)	327 (26.4%)		418 (28.2%)	52 (25.4%)	366 (28.6%)	
	Grade III	118 (8.1%)	20 (9.6%)	98 (7.9%)		131 (8.8%)	13 (6.3%)	118 (9.2%)	
	Grade IV	58 (4.0%)	10 (4.8%)	48 (3.9%)		65 (4.4%)	13 (6.3%)	52 (4.1%)	
	Missing	51	5	46	–	71	6	65	–
Chronic GvHD	No	1041 (78.6%)	159 (83.7%)	882 (77.8%)	–	1119 (80.9%)	143 (77.3%)	976 (81.5%)	–
	Yes	283 (21.4%)	31 (16.3%)	252 (22.2%)		264 (19.1%)	42 (22.7%)	222 (18.5%)	
	Missing	174	23	151	–	173	26	147	–
Extensive cGvHD	No	1166 (89.3%)	173 (91.0%)	993 (89.0%)	–	1232 (90.0%)	161 (87.0%)	1071 (90.5%)	–
	Yes	140 (10.7%)	17 (9.0.%)	123 (11.0%)		136 (10.0%)	24 (13.0%)	112 (9.5%)	
	Missing	192	23	169	–	188	26	162	–
Engraftment	No	10 (0.7%)	4 (1.9%)	6 (0.5%)	–	28 (1.8%)	3 (1.5%)	25 (1.9%)	–
	Yes	1470 (99.3%)	208 (98.1%)	1262 (99.5%)		1505 (98.2%)	204 (98.5%)	1301 (98.1%)	
	Missing	18	1	17	–	23	4	19	–

Variables not shown: age (categorial), diagnosis to HSCT (categorial), donor (sex), and donor to patient sex.

*AraC* cytarabine, *BM* bone marrow, *Bu* busulfan, *CC* chemotherapy-based conditioning, *CI* confidence interval, *CMV* cytomegalovirus, *CR1* first complete remission, *CR2* second complete remission, *Cy* cyclophosphamide, *Eto* etoposide, *Flu* fludarabine, *FTBI* fractionated total body irradiation, *GvHD* graft versus host disease, *HSCT* hematopoietic stem cell transplantation, *IQR* interquartile range, *Mel* melphalan, *MRD* matched-related donor, *PBSC* peripheral blood stem cell, *Thio* thiotepa, *UD* unrelated donor.

^a^CC cohort (*n* = 213) includes: Bu/Cy (*n* = 68), Bu/Cy/Eto (*n* = 66), Bu/AraC/+/−Mel (*n* = 23), Bu/Cy/Mel (*n* = 20), Bu/Flu (*n* = 20), Bu/Cy/Thio (*n* = 14), Bu/Flu/Thio (*n* = 2).

^b^FTBI cohort (*n* = 1285) includes: FTBI/Cy (*n* = 494), FTBI/Eto (*n* = 419), FTBI/other (*n* = 245), FTBI/Cy/Eto (*n* = 78), FTBI/Mel (*n* = 44), FTBI/Cy/Flu (*n* = 5).

^c^CC cohort (*n* = 211) includes: Bu/Cy (*n* = 68), Bu/Cy/Eto (*n* = 52), Bu/AraC/+/−Mel (*n* = 35), Bu/Cy/Thio (*n* = 18), Bu/Cy/Mel (*n* = 17), Bu/Flu (*n* = 13), Bu/Flu/Thio (*n* = 8).

^d^FTBI cohort (*n* = 1345) includes: FTBI/Cy (*n* = 496), FTBI/Eto (*n* = 365), FTBI/other (*n* = 291), FTBI/Cy/Eto (*n* = 91), FTBI/Mel (*n* = 86), FTBI/Cy/Flu (*n* = 16).

### Hematopoietic stem cell donors and source

1.626 patients (53%) were grafted from an UD and 1.428 patients (47%) from a MRD. The majority (*n* = 2.105, 69%) received BM and 949 patients (31%) received PBSC (Table 1).

### Preparative regimens

The most commonly applied conditioning regimens were FTBI-based (*n* = 2.630). In CR1 and CR2, 1.285 (86%) and 1.345 (86%) patients, respectively, received an FTBI-based conditioning. 424 patients received a CC (CR1: *n* = 213 (14%), CR2 *n* = 211 (14%), Table 1).

### FTBI-based

FTBI/Cy (*n* = 990, 38%) and FTBI/Eto (*n* = 784, 30%) were the two most frequent used combinations. The remaining patients received different other FTBI-based combinations (*n* = 856, 32%, Table 1).

### Chemotherapy-based

In the CR1 cohort, 213 patients (14%) received CC. These regimens consisted of Busulfan/Cyclophosphamide (Bu/Cy, *n* = 68), Bu/Cy/Etoposide (Bu/Cy/Eto, *n* = 66), Bu/Cytarabine (AraC)/+/−Melphalan (Mel, *n* = 23), Bu/Cy/Mel (*n* = 20), Bu/Fludarabine (Flu, *n* = 20), Bu/Cy/Thiotepa (Thio, *n* = 14), and Bu/Flu/Thio (*n* = 2).

In the CR2 cohort, 211 patients (14%) received CC. These regimens consisted of Bu/Cy (*n* = 68), Bu/Cy/Eto (*n* = 52), Bu/AraC/+/−Mel (*n* = 35), Bu/Cy/Thio (*n* = 18), Bu/Cy/Mel (*n* = 17), Bu/Flu (*n* = 13), and Bu/Flu/Thio (*n* = 8, Table 1).

### Outcomes

#### Patients transplanted in CR1

**Fig. 1:**
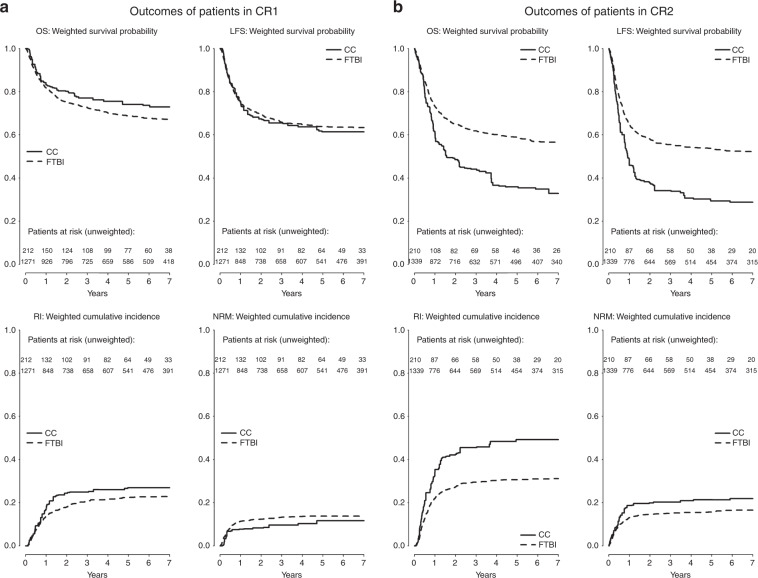
Survival by conditioning regimen. **a** Outcomes of patients in CR1. **b** Outcomes of patients in CR2. CC chemotherapy-based conditioning, CR1 first complete remission, CR2 second complete remission, FTBI fractionated total body irradiation, LFS leukemia-free survival, NRM nonrelapse mortality, OS overall survival, RI relapse incidence.

Five years OS was 68.8% (95% CI 66.3–71.5) after FTBI and 74.1% (95% CI 71.1–77.3) after CC (*P* = 0.25). Five years LFS was 63.8% (95% CI 61.2–66.5) after FTBI and 61.4% (95% CI 58.0–64.9) after CC (*P* = 0.83). Five years RI was 22.4% (95% CI 20.1–25.0) after FTBI and 26.9% (95% CI 19.7–36.9) after CC (*P* = 0.33). Five years NRM was 13.8% (95% CI 11.9–15.9) after FTBI and 11.7% (95% CI 6.9–19.8) after CC (*P* = 0.47). Incidence of aGvHD grade III–IV at day 100 was 11.8% (95% CI 10.1–13.7) after FTBI and 16.9% (95% CI 10.7–26.7) after CC (*P* = 0.16). Five years incidence of cGvHD was 24.3% (95% CI 21.8–27.1) after FTBI and 20.8% (95% CI 13.7–31.4) after CC (*P* = 0.60). Five years incidence of extensive cGvHD was 11.3% (95% CI 9.5–13.4) after FTBI and 8.2% (95% CI 4.3–15.9) after CC (*P* = 0.54, Table 2, Fig. 1a).

**Table 2:** Weighted analysis of survival by conditioning regimen of patients in CR1 and CR2.

Outcome	Strata	*n*	Day 100 (95% CI)	1 year (95% CI)	3 years (95% CI)	5 years (95% CI)	HR	95% CI	*P* value*
CR1 (*n* = 1483)									
OS	FTBI	1271	–	81.8 (79.7–83.8)	72.5 (70.1–75.0)	68.8 (66.3–71.5)	1.00	–	0.25
	CC	212	–	83.2 (80.7–85.8)	77.1 (74.2–80.0)	74.1 (71.1–77.3)	0.79	0.53–1.17	
LFS	FTBI	1271	–	74.7 (72.4–77.0)	65.8 (63.3–68.5)	63.8 (61.2–66.5)	1.00	–	0.83
	CC	212	–	75.4 (72.5–78.4)	65.5 (62.3–68.9)	61.4 (58.0–64.9)	1.03	0.76–1.41	
RI	FTBI	1271	–	13.9 (12.1–16.1)	20.9 (18.7–23.4)	22.4 (20.1–25.0)	1.00	–	0.33
	CC	212	–	16.9 (11.3–25.3)	24.9 (17.9–34.5)	26.9 (19.7–36.9)	1.20	0.83–1.71	
NRM	FTBI	1271	–	11.4 (9.7–13.3)	13.3 (11.5–15.4)	13.8 (11.9–15.9)	1.00	–	0.47
	CC	212	–	7.7 (4.1–14.7)	9.6 (5.5–17.0)	11.7 (6.9–19.8)	0.79	0.41–1.50	
aGvHD III–IV	FTBI	1225	11.8 (10.1–13.7)	–	–	–	1.00	–	0.16
	CC	207	16.9 (10.7–26.7)	–	–	–	1.46	0.86–2.50	
cGvHD	FTBI	1123	–	20.0 (17.6–22.6)	23.3 (20.9–26.1)	24.3 (21.8–27.1)	1.00	–	0.60
	CC	189	–	18.5 (11.8–29.1)	20.8 (13.7–31.4)	20.8 (13.7–31.4)	0.86	0.50–1.48	
cGvHD ext.	FTBI	1107	–	8.2 (6.7–10.0)	10.4 (8.7–12.4)	11.3 (9.5–13.4)	1.00	–	0.54
	CC	189	–	6.5 (3.1–13.8)	8.2 (4.3–15.9)	8.2 (4.3–15.9)	0.82	0.42–1.58	
CR2 (*n* = 1549)									
OS	FTBI	1339	–	73.1 (70.8–75.4)	61.9 (59.4–64.5)	58.8 (56.2–61.6)	1.00	–	**<0.0001**
	CC	210	–	61.0 (58.1–64.1)	44.1 (41.1–47.4)	35.9 (33.0–39.1)	1.75	1.36–2.27	
LFS	FTBI	1339	–	65.1 (62.6–67.6)	55.5 (52.9–58.2)	53.7 (51.1–56.5)	1.00	–	**<0.0001**
	CC	210	–	45.9 (43.0–49.1)	34.2 (31.3–37.3)	29.4 (26.6–32.5)	1.80	1.43–2.25	
RI	FTBI	1339	–	21.9 (19.7–24.3)	29.5 (27.0–32.2)	30.6 (28.1–33.3)	1.00	–	**<0.0001**
	CC	210	–	35.4 (27.3–45.9)	45.6 (36.8–56.5)	49.3 (40.3–60.2)	1.96	1.44–2.67	
NRM	FTBI	1339	–	13.0 (11.3–15.0)	15.0 (13.2–17.2)	15.7 (13.8–17.9)	1.00	–	**0.044**
	CC	210	–	18.7 (12.7–27.4)	20.2 (14.1–29.0)	21.3 (15.1–30.2)	1.51	1.01–2.25	
aGvHD III–IV	FTBI	1274	13.1 (11.4–15.1)	–	–	–	1.00	–	0.36
	CC	204	10.6 (6.5–17.2)	–	–	–	0.80	0.51–1.28	
cGvHD	FTBI	1192	–	17.9 (15.7–20.4)	20.5 (18.1–23.1)	21.2 (18.8–23.9)	1.00	–	0.064
	CC	184	–	25.3 (17.7–36.2)	25.8 (18.1–36.7)	26.0 (18.3–36.9)	1.52	0.98–2.37	
cGvHD ext.	FTBI	1178	–	7.5 (6.1–9.3)	9.6 (8.0–11.5)	9.9 (8.3–11.9)	1.00	–	0.075
	CC	184	–	9.5 (5.7–16.1)	10.0 (6.0–16.5)	10.1 (6.2–16.7)	1.64	0.95–2.84	

*a/cGvD* acute/chronic graft versus host disease, *CC* chemotherapy-based conditioning, *CI* confidence interval, *CR1* first complete remission, *CR2* second complete remission, *ext.* extensive, *FTBI* fractionated total body irradiation, *HR* Hazard ratio, *LFS* leukemia-free survival, *NRM* nonrelapse mortality, *OS* overall survival, *RI* relapse incidence.

*Wald test in a weighted Cox model taking into account the center effect (cause specific for RI, NRM, and GvHD outcomes).

#### Patients transplanted in CR2

FTBI was superior compared with CC in terms of OS, LFS, RI, and NRM. In detail, five years OS was 58.5% (95% CI 56.2–61.6) after FTBI and 35.9% (95% CI 33.0–39.1) after CC (*P* < 0.0001). Five years LFS was 53.7% (95% CI 51.1–56.5) after FTBI and 29.4% (95% CI 26.6–32.5) after CC (*P* < 0.0001). Five years RI was 30.6% (95% CI 28.1–33.3) after FTBI and 49.3% (95% CI 40.3–60.2) after CC (*P* < 0.0001). Five years NRM was 15.7% (95% CI 13.8–17.9) after FTBI and 21.3% (95% CI 15.1–30.2) after CC (*P* = 0.044).

Significant differences in the incidence of aGvHD grade III–IV at day 100, cGvHD and extensive cGvHD were not detected (Table 2, Fig. 1b).

## Discussion

Most pediatric patients with ALL aged above 2 years who undergo allo-HSCT receive FTBI as part of the preparative regimen [1–5, 8–10, 12, 13, 15–17]. Adverse late effects such as endocrine disorders, infertility, cognitive impairment, cataracts, and increased risk for secondary malignancies, are a major burden of this treatment modality but can at least to a certain extent also occur after CC (e.g., Bu/Cy/Eto) [18–22]. However, to date, it has not been proven whether FTBI could be advantageously omitted from the preparation for allo-HSCT and replaced by CC without jeopardizing LFS [3, 5, 24, 25, 34]. Nevertheless, myeloablative CC remains widely applied in Europe and elsewhere. To compare outcomes of FTBI with CC in pediatric ALL, we performed this multinational retrospective study.

Our study cohort has been intentionally restricted to patients having received first allo-HSCT in CR1 or CR2 after MAC, BM or PBSC as stem cell source from MRD or UD as donors in order to receive a more uniform cohort. In this study, all CC regimens were Bu-based. Bu/Cy, a well-established preparative regimen for pediatric [35–37] and adult patients [38, 39], was most frequently applied. Bu/Cy/Eto was the second most frequently used MAC. This combination was applied in the international Berlin–Frankfurt–Münster (iBFM/BFM) clinical trials [1, 40, 41], particularly in infants (Interfant-99) [42, 43], and elsewhere [2, 44–47]. Within the observation period of this study, the alternative alkylator, treosulfan, was increasingly used for children with malignancies; but no treosulfan-containing regimen reached a significant number of cases [48].

The FTBI and the CC group differed with regard to number of cases, as well as some clinical features, as mentioned above (see Statistical analysis). These potential confounders have been adjusted by the inverse IPW method (propensity score) in order to allow the comparison of the outcomes of the two conditioning groups.

In the CR1 cohort the outcome after FTBI was not significantly different compared with CC. This is a new, interesting finding; although we do not know the reasons for omission of FTBI, which could be manifold: (1) young age, (2) negativity of minimal residual disease before allo-HSCT, (3) high risk for toxicity and infection after having experienced complications during front line therapy, (4) logistical reasons as no access to timely FTBI, and (5) decision of patients/parents. However, due to the large number of participating centers from various countries, there might be some equipoise.

Not surprisingly, overall outcomes of the CR2 cohort were inferior compared with CR1 patients. This was predominantly attributed to a significantly higher RI in the CC group of the CR2 cohort. It was impossible to evaluate risk factors for this difference. One could speculate, that patients with increased risk for toxicity due to pretransplant complications and/or a history of cranial/spinal irradiation were stratified to an irradiation-free conditioning.

Interestingly, outcomes after FTBI were superior as compared with CC with regard to OS, LFS, RI, and NRM in our CR2 cohort. More importantly, superior OS, LFS, and RI of the FTBI cohort did not result in a higher but even a lower NRM compared with the CC cohort.

Various hypotheses concerning the different impact of FTBI on conditioning of ALL patients in CR1 versus CR2 can be made. One could speculate that:

1. In a retrospective study there is no possibility to identify the background of the decision for the given conditioning. Patients in CR1 with unfavorable prognostic factors might have been conditioned with FTBI and those children with a more favorable risk profile might have been treated with CC. This hypothesis might similarly fit to patients in CR2.
2. Patients in CR2 had more often extra medullary leukemia and would benefit from FTBI.
3. Relapsed ALL was more resistant to chemotherapeutic agents and benefited from FTBI as a new treatment element.

The potential superiority of FTBI-based conditioning in pediatric ALL was also demonstrated in literature. In 2000, Davies et al. reported a 3-year LFS of 50% after FTBI versus 35% after Bu-based conditioning (*P* = 0.005) in a cohort of 627 pediatric patients mainly transplanted in CR1 and CR2 [26]. Three years later, Bunin et al. found a 3-year EFS of 58% after FTBI versus 29% after Bu-based conditioning (*P* = 0.03) in a randomized cohort of 43 children, transplanted in CR1-3 [2].

The main merit of our study is that it includes a large cohort of pediatric ALL patients who, while in remission, received FTBI as well as myeloablative CC for first allo-HSCT using BM and PBSC from MRD and UD following to several European protocols [39, 40]. Consequently, this retrospective study represents “real-world practice.” On the other hand, this registry-based study has some limitations resulting in the fact that our results must be considered as preliminary. In fact, no data were available on: (1) The administration mode of Bu (intravenous or oral) or use of therapeutic drug monitoring and dose adjustment. (2) Cytogenetics or molecular genetics of ALL. (3) Toxicity or reasons for NRM. (4) Secondary malignancies. (5) Minimal residual disease levels at time of allo-HSCT. (6) Site of relapse after front line ALL therapy or after allo-HSCT. (7) CNS involvement. (8) Date of relapse for patients in CR2. The latter information is necessary for classifying a relapse event as very early, early or late for further patient stratification in classes of risk [49], and for a more detailed analysis of the survival of patients transplanted in CR2. Furthermore, since our retrospective study cohort included B- as well as T-ALL phenotypes, BM and PBSC as stem cell sources, MRD and UD, only children above 2 years of age and spanned an observation time of 13 years, our study population still has a heterogeneous character. Moreover, our non-FTBI-receiving CC cohort is relatively small compared with the FTBI group.

We conclude that FTBI-based conditioning was superior to CC in terms of OS, LFS, RI, and NRM for children undergoing allo-HSCT in CR2, according to the largest study comparing outcomes of FTBI versus CC for first allo-HSCT in pediatric ALL. However, we must stress the preliminary character of the results of this retrospective “real-world-practice” study.

Prospective data comparing FTBI and CC for allo-HSCT in children and adolescents with ALL are urgently needed. Due to the limitations of retrospective studies, it seemed justified to ask whether CC is at least as effective as a FTBI-based conditioning in terms of outcome, toxicity, and late effects in a prospective, preferably randomized clinical trial. The answer to this relevant question will be hopefully obtained by the prospective international, multicenter ALL SCTped 2012 FORUM (“For Omitting Radiation Under Majority Age”) randomized trial, which was initiated in 2012 (EudraCT number: 2012-003032-22).
