# Correction: Myeloablative conditioning for allo-HSCT in pediatric ALL: FTBI or chemotherapy?—A multicenter EBMT-PDWP study

**DOI:** 10.1038/s41409-021-01424-5

**Published:** 2021-08-19

**Authors:** Andre Manfred Willasch, Christina Peters, Petr Sedláček, Jean-Hugues Dalle, Vassiliki Kitra-Roussou, Akif Yesilipek, Jacek Wachowiak, Arjan Lankester, Arcangelo Prete, Amir Ali Hamidieh, Marianne Ifversen, Jochen Buechner, Gergely Kriván, Rose-Marie Hamladji, Cristina Diaz-de-Heredia, Elena Skorobogatova, Gérard Michel, Franco Locatelli, Alice Bertaina, Paul Veys, Sophie Dupont, Reuven Or, Tayfun Güngör, Olga Aleinikova, Sabina Sufliarska, Mikael Sundin, Jelena Rascon, Ain Kaare, Damir Nemet, Franca Fagioli, Thomas Erich Klingebiel, Jan Styczynski, Marc Bierings, Kálmán Nagy, Manuel Abecasis, Boris Afanasyev, Marc Ansari, Kim Vettenranta, Amal Alseraihy, Alicja Chybicka, Stephen Robinson, Yves Bertrand, Alphan Kupesiz, Ardeshir Ghavamzadeh, Antonio Campos, Herbert Pichler, Arnaud Dalissier, Myriam Labopin, Selim Corbacioglu, Adriana Balduzzi, Jacques-Emmanuel Galimard, Peter Bader, on behalf of the EBMT Paediatric Diseases Working Party

**Affiliations:** 1 Division for Stem Cell Transplantation and Immunology, Department for Children and Adolescents, University Hospital, Goethe University Frankfurt, Frankfurt, Germany; 2 St. Anna Children’s Hospital, Department of Pediatrics, Medical University of Vienna, Vienna, Austria; 3 Department of Pediatric Hematology and Oncology, University Hospital Motol, Prague, Czech Republic; 4 Department of Pediatric Hemato–Immunology, Hôpital Robert Debré and Université de Paris, Paris, France; 5 Stem Cell Transplant Unit, Aghia Sophia Children’s Hospital, Thivon and Papadiamantopoulou, Athens, Greece; 6 Department of Pediatric Hematology and Stem Cell Transplantation Unit, Medicalpark Antalya Hastanesi, Antalya, Turkey; 7 Department of Pediatric Oncology, Hematology and Transplantology, University of Medical Sciences, Poznan, Poland; 8 Department of Pediatrics, Leiden University Medical Center, Leiden, The Netherlands; 9 Hematology–Oncology Unit “Lalla Seràgnoli”, Department of Pediatrics, University of Bologna, Bologna, Italy; 10 Pediatric Cell Therapy Research Center, Tehran University of Medical Sciences, Tehran, Iran; 11 Department of Pediatrics, Copenhagen University Hospital, Rigshospitalet, Copenhagen, Denmark; 12 Department of Pediatric Hematology and Oncology, Oslo University Hospital, Oslo, Norway; 13 Department for Pediatric Hematology and Hemopoietic Stem Cell Transplantation, Central Hospital of Southern Pest, National Institute of Hematology and Infectious Diseases, United St. Istvan and St. László Hospital, Budapest, Hungary; 14 Centre Pierre et Marie Curie, Service Hématologie Greffe de Moelle, Alger, Algeria; 15 Servicio de Hematologia y Oncologia Pediátricas, Hospital Universitario Vall d’Hebron, Barcelona, Spain; 16 BMT Department, Russian Children’s Hospital, Moscow, Russia; 17 Department of Pediatric Hematology and Oncology and Research Unit EA 3279, Timone Enfants Hospital, AP-HM and Aix-Marseille University, Marseille, France; 18 Dipartimento di Onco-Ematologia e Terapia Cellulare e Genica, IRCCS Ospedale Pediatrico Bambino Gesù, Rome, Italy; 19 Dipartimento Materno-Infantile e Scienze Urologiche, Sapienza University of Rome, Rome, Italy; 20 Division of Stem Cell Transplantation and Regenerative Medicine, Department of Pediatrics, School of Medicine, Stanford University, Stanford, CA USA; 21 Great Ormond Street Hospital for Children NHS Foundation Trust, London, UK; 22 Department of Pediatric Hematology and Oncology, Cliniques Universitaires Saint-Luc, Brussels, Belgium; 23 Department of Bone Marrow Transplantation, Hadassah-Hebrew University Medical Center, Jerusalem, Israel; 24 Division of Stem Cell Transplantation, Children’s Research Center (CRC), University Children’s Hospital, Zurich, Switzerland; 25 Republic Clinical Research Centre for Pediatric Oncology and Hematology, Minsk, Belarus; 26 BMT Unit, Department of Pediatric Hematology and Oncology, Comenius University Medical School, Limbová, Bratislava, Slovak Republic; 27 Division of Pediatrics, CLINTEC, Karolinska Institutet and Pediatric Hematology, Immunology and HCT, Astrid Lindgren Children’s Hospital, Karolinska University Hospital, Stockholm, Sweden; 28 Center of Pediatric Oncology and Hematology, Vilnius University, Vilnius, Lithuania; 29 Tartu University Hospital, Tartu, Estonia; 30 Department of Haematology, Internal Clinic, University Hospital Centre, Zagreb, Croatia; 31 Paediatric Onco-Haematology, City of Science and Health of Turino, Regina Margherita Children’s Hospital, Torino, Italy; 32 Department of Pediatric Hematology and Oncology, Collegium Medicum, Nicolaus Copernicus University Torun, Bydgoszcz, Poland; 33 BMT-Unit, University Medical Centre Utrecht Pediatrics, Utrecht, The Netherlands; 34 Department of Hematology, Child Welfare Center, Borsod County Teaching Hospital, Miskolc, Hungary; 35 Bone Marrow Transplant Program, Instituto Portugues Oncologia, Lisbon, Portugal; 36 Raisa Gorbacheva Memorial Research Institute for Pediatric Oncology, Haematology and Transplantation, Saint Petersburg State Medical I.P. Pavlov University, Saint Petersburg, Russia; 37 Department of Pediatrics, Onco-Hematology Unit, Geneva University Hospital, Geneva University, Geneva, Switzerland; 38 Hospital for Children and Adolescents, University of Helsinki, Helsinki, Finland; 39 Department of Pediatric Hematology Oncology, King Faisal Specialist Hospital & Research Center, Riyadh, Saudi Arabia; 40 Department of Bone Marrow Transplantation, Oncology and Hematology, Cape of Hope Medical Center, Wroclaw Medical University, Wroclaw, Poland; 41 University Hospitals Bristol NHS Foundation Trust, Bristol, UK; 42 Department of Pediatric Hematology and BMT, IHOP and Claude Bernard University, Lyon, France; 43 Department of Pediatric Hematology–Oncology, School of Medicine, Akdeniz University, Antalya, Turkey; 44 Hematology-Oncology and BMT Research, Shariati Hospital, Tehran, Iran; 45 Bone Marrow Transplantation Service, Portuguese Institute of Oncology, Porto, Portugal; 46 EBMT Paediatric Diseases Working Party, Paris, France; 47 EBMT Paris Study Office, Paris, France; 48 Department of Pediatric Hematology, Oncology and Stem Cell Transplantation, University of Regensburg, Regensburg, Germany; 49 Bone Marrow Transplantation Unit, Pediatric Department of Milano-Bicocca University, Fondazione Monza e Brianza per il Bambino e la sua Mamma Foundation, Monza, Italy

**Keywords:** Stem-cell research, Acute lymphocytic leukaemia

Correction to: *Bone Marrow Transplantation* 10.1038/s41409-020-0854-0, published online 17 March 2020

The article “Myeloablative conditioning for allo-HSCT in pediatric ALL: FTBI or chemotherapy?—A multicenter EBMT-PDWP study,” written by Andre Manfred Willasch, Christina Peters, Petr Sedláček, Jean-Hugues Dalle, Vassiliki Kitra-Roussou, Akif Yesilipek, Jacek Wachowiak, Arjan Lankester, Arcangelo Prete, Amir Ali Hamidieh, Marianne Ifversen, Jochen Buechner, Gergely Kriván, Rose-Marie Hamladji, Cristina Diaz-de-Heredia, Elena Skorobogatova, Gérard Michel, Franco Locatelli, Alice Bertaina, Paul Veys, Sophie Dupont, Reuven Or, Tayfun Güngör, Olga Aleinikova, Sabina Sufliarska, Mikael Sundin, Jelena Rascon, Ain Kaare, Damir Nemet, Franca Fagioli, Thomas Erich Klingebiel, Jan Styczynski, Marc Bierings, Kálmán Nagy, Manuel Abecasis, Boris Afanasyev, Marc Ansari, Kim Vettenranta, Amal Alseraihy, Alicja Chybicka, Stephen Robinson, Yves Bertrand, Alphan Kupesiz, Ardeshir Ghavamzadeh, Antonio Campos, Herbert Pichler, Arnaud Dalissier, Myriam Labopin, Selim Corbacioglu, Adriana Balduzzi, Jacques-Emmanuel Galimard, Peter Bader, on behalf of the EBMT Paediatric Diseases Working Party, was originally published online first without Open Access. After publication in volume 55, issue 8, page 1540–1551, the author decided to opt for Open Choice and to make the article an Open Access publication. Therefore, the copyright of the article has been changed to © The Author(s) 2020 and the article is forthwith distributed under the terms of the Creative Commons Attribution 4.0 InternationalS License, which permits use, sharing, adaptation, distribution, and reproduction in any medium or format, as long as you give appropriate credit to the original author(s) and the source, provide a link to the Creative Commons license, and indicate if changes were made. The images or other third-party material in this article are included in the article’s Creative Commons license, unless indicated otherwise in a credit line to the material. If material is not included in the article’s Creative Commons license and your intended use is not permitted by statutory regulation or exceeds the permitted use, you will need to obtain permission directly from the copyright holder. To view a copy of this license, visit http://creativecommons.org/licenses/by/4.0. Open access funding was enabled and organized by Projekt DEAL.

